# Trends in the Use of Low and No-Calorie Sweeteners in Non-Alcoholic Beverages in Slovenia

**DOI:** 10.3390/foods10020387

**Published:** 2021-02-10

**Authors:** Edvina Hafner, Maša Hribar, Hristo Hristov, Anita Kušar, Katja Žmitek, Mark Roe, Igor Pravst

**Affiliations:** 1 Nutrition Institute, Tržaška Cesta 40, SI-1000 Ljubljana, Slovenia; edvina.hafner@nutris.org (E.H.); masa.hribar@nutris.org (M.H.); hristo.hristov@nutris.org (H.H.); anita.kusar@nutris.org (A.K.); katja.zmitek@vist.si (K.Ž.); 2 VIST—Higher School of Applied Sciences, Gerbičeva Cesta 51A, SI-1000 Ljubljana, Slovenia; 3 EuroFIR AISBL, 1050 Brussels, Belgium; mr@eurofir.org; 4 Biotechnical Faculty, University of Ljubljana, Jamnikarjeva 101, SI-1000 Ljubljana, Slovenia

**Keywords:** low- and no-calorie sweeteners, non-alcoholic beverages, added sugar, food supply, food labelling

## Abstract

Excessive sugar intake and the associated increased obesity risk indicate that food reformulation is needed. Non-alcoholic beverages are often high in free sugars, making reformulation with low and no-calorie sweeteners (LNCS) a common choice. Our knowledge of the use of LNCS in the European food supply is, however, very limited. This study aimed to evaluate the trends in the use of LNCS in different non-alcoholic beverages in the Slovenian food supply over the course of two years. We assessed which LNCS are most used and how the use of LNCS affects energy and sugar content. We analyzed labeling information of non-alcoholic beverages in selected grocery stores, covering the majority of the Slovenian food supply. Selected grocery stores were located in the capital city (Ljubljana). LNCS were present in 13.2% and 15.5% of non-alcoholic beverages in the years 2017 and 2019, respectively. The use of LNCS has significantly increased only in energy drinks (*p* < 0.01). The most used LNCS in 2017 were acesulfame K, aspartame, and cyclamates. In 2019 the use of sucralose increased significantly (*p* < 0.01) to become the second most used LNCS. Energy and sugar content varied between subcategories, which depended on the presence of added sugar and LNCS. Comparison between the energy value and the presence of the LNCS showed an almost 50% lower energy content of beverages containing both added sugar and LNCS (E_2017_ = 92.8 kJ; E_2019_ = 96.2 kJ per 100 mL), compared with beverages with only added sugar (E_2017_ = 161.8 kJ; E_2019_ = 159.0 kJ per 100 mL). In beverages sweetened only with LNCS, the difference was even more noticeable (E_2017_ = 22.3 kJ; E_2019_ = 14.3 kJ per 100 mL). Results show that the use of LNCS can help producers reduce the energy value of non-alcoholic beverages. Still, compared to other countries, the offer of such products in the Slovenian food supply is relatively low. However, due to possible public health risks of excessive use of LNCS, producers should be further encouraged for reformulation and production of less sweet products without LNCS, enabling consumers to adapt to less sweet taste of beverages. Further monitoring of LNCS in the food supply is therefore recommended, preferably also with consideration of sales data.

## 1. Introduction

Diet-related risk factors, including obesity/overweight, have been associated with some non-communicable diseases such as cardiovascular diseases, diabetes mellitus and certain cancers. One of the main problems in the modern diet is the consumption of excessive amounts of sugar [1]. Term total sugars include all mono- and disaccharides, while free and added sugars exclude naturally occurring sugars in milk, fruit, and vegetables. The main difference between the latter groups is that free sugars, unlike added sugars, include sugars from fruit purees and juices. The relation between excessive intake and negative health outcomes is most consistent for free sugars, which negatively affects both the health of the oral cavity and risks for the development of chronic diseases [2,3]. In 2003 the World Health Organization (WHO) issued a recommendation that free sugar intake should not exceed 10% of total energy intake [4]. The WHO updated their guidelines in 2015, suggesting further reduction of the intake of free sugars to below 5% of total energy intake [5].

Non-alcoholic beverages are often high in free sugars and acids and often supply only energy with little or no nutritional benefit. The consumption of sugar-containing beverages increased dramatically over the past couple of decades, which has been suggested as one of the major contributors to the obesity epidemic [6]. Despite the known negative effects, the consumption of sugar-sweetened beverages is high, especially in children and adolescents [7]. Many national health organizations have implemented public health initiatives for sugar reduction, and some countries also introduced regulatory interventions, such as a tax on sugary beverages [8]. Legal restrictions and increased awareness have encouraged producers to offer alternative beverages with reduced sugar content. However, reducing the amount of sugar in beverages can have a negative impact on sensory perception, affecting product sales. Many manufacturers therefore offer beverages with low and no-calorie sweeteners (LNCS) [1].

LNCS are regulated food additives that provide a sweet taste without high energy and sugar content [9]. LNCS include sweeteners that have lower (polyols) or no (intensive sweeteners) energy value. Intensive sweeteners have high sweetness potency and do not contribute to the energy value due to the low amount used for desired taste. They can be of synthetic or natural origin, and they all have assigned acceptable daily intake (ADI). Polyols have sweetness potency similar to sucrose, but lower energy value (10 kJ per gram) making them easy replacement when considering the volume of the product. A warning is needed when their content in product is higher than 10%, as they have a laxative effect [10,11]. There are 19 LNCS approved for use in Europe: acesulfame K, aspartame, cyclamates, isomalt, saccharin, sucralose, thaumatin, neohesperidin DC, steviol glycosides, neotame, aspartame-acesulfame salt, advantame, sorbitol, mannitol, polyglycitol syrup, maltitols, lactitol, xylitol, and erythritol [10].

LNCS may not promote health, but they are particularly useful for providing sweet taste to individuals who need to avoid sugar intake because of medical condition, e.g., diabetics [12]. It should be noted that the proportion of such people is growing both globally and nationally. For example, the prevalence of diagnosed diabetes in Slovenia increased by 29% from 2009 to 2018 [13], while more than half of the Slovenian population is overweight or obese [14]. The growth of these diseases and recommendations to reduce sugar intake are increasing the number of potential consumers. Therefore, it is not surprising that both the supply of such products and the consumption of LNCS in the general population are also growing in some countries [15].

It should be noted that, from all types of foods, beverages tend to have the greatest benefits from using LNCS, because sugar is usually their main or only source of energy [16]. Therefore, non-alcoholic beverages have already been shown to be a major source of LNCS [17,18], which raises questions about whether beverages with LNCS may also have negative effects on health. The use of LNCS is carefully regulated, on the basis of scientific opinions or different organizations, such as the European Food Safety Authority (EFSA), the Food and Drug Administration (FDA) and the WHO. According to available safety assessments, dietary intakes of LNCS below ADI, should not provide health risks for the consumer [19,20]. However, some researchers highlighted the potential adverse effects of excessive use of LNCS on the gut microbiome, body weight, diabetes development, and other metabolic conditions [21,22]. The safety of chronic consumption of larger quantities of LNCS throughout life is also not well researched. This could be problematic for some diabetics who have been consuming sweeteners regularly for decades [17]. Another point of concern is the possibility for exceeding ADI levels in children, where the quantity of consumed soft drinks can be high, while their body mass is low.

Monitoring food reformulation is challenging, even when only focusing on specific nutrients (i.e., sugar content [23]), but even more challenging when other food constituents need to be considered. While the content of sugar and some other nutrients are provided on food labels in a very structured way [24], food additives and other constituents are provided only in ingredient lists as qualitative information, making analyses more difficult. Methodology for monitoring ingredients in the food supply was developed within the Global Food Monitoring Group initiative and INFORMAS network [25]. A recommended approach, which was used in this study, is the use of snap-shot datasets collected in food markets. More recently, the European Union’s Horizon 2020 project Food Nutrition Security Cloud project (FNS-Cloud; https://www.fns-cloud.eu/ (accessed on 9 February 2021)) was launched, with the aim of enabling interoperability and standardization of data. This will help overcome data fragmentation, allowing centralized access to existing and emerging datasets and the development of new services to support the use of available data. The exploitation of alternative data sources (including crowd-sourced data) and development and testing of tools, that will make use of food labelling data more efficient, is also an essential part of the project. This part of the project focuses not only on the nutritional composition of foods but also on the use of information on nonnutritive food additives.

Data on food additives such as LNCS are limited. While consumers seek alternative products with lower energy content and sugar density, a better understanding of the use of LNCS in the food supply is essential [26]. This study was aimed to evaluate the prevalence of LNCS in different types of non-alcoholic beverages in the Slovenian food supply over the course of two years. We assessed which LNCS are most used and examined the relationship between the presence of LNCS, and energy and sugar content. Study results were compared with similar studies abroad in order to see the differences between countries, providing insights of the future trends of the use of the LNCS in the food supply.

## 2. Materials and Methods

### 2.1. Data Collection and Categorization

To provide insights on the trends of using LNCS, the study was conducted using a food supply sample for the years 2017 and 2019. The source of data was the Composition and Labelling Information System (CLAS) database of the Nutrition Institute (Ljubljana, Slovenia) [27], which contains information on prepacked foods in the Slovenian food supply. This database is composed of food-labelling data for branded foods, which were available at the time of sampling in selected grocery stores in Slovenia. Samples included major retailers in Slovenia, which represent the majority of the market share for branded foods. The data collection process is described in detail elsewhere [23,28]. In short, the data was collected in stores of five different retailers (Interspar Vič, Mercator Šiška, Tuš Vič, and Hofer Brdo and Lidl Bežigrad), all located in Ljubljana, the capital city of Slovenia. With the permission of retailers, we systematically photographed all available prepacked products with a unique European Article Number (EAN) barcode and collected them in the CLAS database. All product information was extracted from photographs.

The categorization of products was made according to the classification developed by Dunford et al. [25]. Minor changes were made according to the specifics of the European market. The focus was on the beverage category, in which we selected the following subcategories: juices, nectars, energy drinks, sports drinks and soft drinks. Products in each subcategory were also classified as containing LNCS and/or added sugar, based on their food labelling information.

The subcategories of juices and nectars included products according to local regulatory definition. Subcategory of juices included only 100% fruit juices, while nectars included beverages with mandatory percentage of fruit between 25% and 50% (depending on the type of fruit) [29]. Isotonic, hypotonic, and hypertonic beverages and beverages which packaging clearly stated are intended for athletes, were included in the subcategory of sports drinks. Energy drinks included all products that were clearly defined as energy drink on the label and that supplied a boost of energy from caffeine, guarana or taurine. Soft drinks included other non-alcoholic flavored beverages, which may be carbonated or non-carbonated, such as colas, tonics, iced teas, fruit drinks and flavored waters. Beverage-type products that need further preparation (i.e., powders for the preparation of drinks or cordials) were excluded from the study. Our total sample included 2264 non-alcoholic beverages (N = 1043 and 1221 for the years 2017 and 2019, respectively).

For statistical evaluation, we used food labelling information about the presence of specific LNCS and added sugar, total sugar and energy content. It should be noted that total sugar content was used for statistical analysis because free sugars are not required on the label. But, due to the nature of the product, most sugars are in free form.

### 2.2. Data Processing and Statistical Analyses

Data about the composition of non-alcoholic beverages were processed using Microsoft SQL Server Management Studio 13.0, Microsoft Analysis Services Client Tools 13.0, Microsoft Data Access Components (MDAC) 10.0, Microsoft Excel 2019 (Microsoft, Redmond, Washington, DC, USA) and the Composition and Labelling Information System (CLAS) (Nutrition Institute, Ljubljana, Slovenia). Statistical analyses were performed using IBM SPSS v.26 (IBM Corp., Armonk, New York, USA).

Descriptive analysis was used to establish the proportion of beverages (for all the selected subcategories) that contained LNCS and the frequency of the use of individual LNCS. The 95% confidence interval was calculated using the Wilson score interval [30]. Two-tailed z-test was used to identify significant changes between the years 2017 and 2019. The subcategory of juices was excluded from this part of the statistics due to the absence of LNCS use in both years. Energy values and total sugar content for each subcategory was presented as means with calculated standard deviation. Comparison of means between 2017 and 2019 was performed using t-test for two independent samples. In all statistical tests, the level of significance was set at *p* < 0.05.

## 3. Results

The sample consisted of 1043 beverages in the year 2017 and 1221 beverages in 2019. Among the selected categories of non-alcoholic beverages, the largest share in the sample had soft drinks, representing approximately half of the sample (2017: 53.2%; 2019: 49.2%). Juices and nectars also contributed a large share to the sample (approximately 40%), while energy and sports drinks comprised the remainder (Table 1).

In 2017 13.2% (N = 138) of non-alcoholic beverages contained at least one LNCS. In 2019 the percentage of beverages with LNCS increased to 15.5% (N = 189), but the difference was not statistically significant (*p* = 0.12). The only statistically significant difference between 2017 and 2019 was in the category of energy drinks, where the percentage of beverages with LNCS increased from 16.9% to 41.8% (*p* < 0.01). Energy drinks were also one of the categories with the highest percentage of products with LNCS. The LNCS were predominantly used in sports drinks (2017: 85.7%; 2019: 63.6%), but it should be noted that this product category contained quite a small sample of products (21 and 22 in years 2017 and 2019, respectively). Overall, the sample contained 327 samples of beverages with added LNCS (138 and 189 in the years 2017 and 2019, respectively). These were mostly soft drinks, as their availability on the market is extensive and they have a relatively high proportion of products with LNCS (2017: 16.8%; 2019: 19.6%). Nectars had a low proportion of products with LNCS (2017: 11.9%; 2019: 7.0%), while, as expected, in juices we did not observe the use of LNCS or added sugar (Table 1).

We observed that majority of the beverages with LNCS contained more than one sweetener (2017: 71.0%; 2019: 61.4%) (Table 2). Only sucralose and steviol glycosides were used as sole sweetener. All other LNCS appeared mostly in mixtures with different LNCS. The most used LNCS in the year 2017 were acesulfame K (64.5%), aspartame (54.3%) and cyclamates (34.8%). In 2019 the use of sucralose increased significantly (*p* < 0.01), while the use of aspartame declined (*p* < 0.01). Therefore, the most common LNCS in the year 2019 was still acesulfame K (55.6%), followed by sucralose (38.1%) and aspartame (33.9%). Changes in proportions of LNCS were mainly due to the significant changes in the use of sucralose and aspartame, which also had an impact on the increased use of a sole sweetener. We observed that sucralose use increased particularly in energy drinks where almost all new products in 2019 contained sucralose. In 2019 two LNCS were identified, which were not found in the 2017 sample, but they were used only in a very few products (neohesperidin DC in five products, and xylitol in one)

We also performed a comparison of mean energy and sugar content for the selected categories, based on the presence of added sugar and LNCS (Table 3). Beverages in each category were divided into four groups, considering the presence of added sugar, added LNCS, both or none of them. The highest mean energy value was observed in beverages with no added sugar and no added LNCS (E_2017_: 174.8 kJ/100 mL; E_2019_: 174.9 kJ/100 mL), the reason for this was that this category was mostly represented by 100% fruit juices, which are naturally high in sugar. A slightly lower mean energy value (E_2017_: 161.8 kJ/100 mL; E_2019_: 159.0 kJ/100 mL) was observed in beverages with added sugar, which were also the most represented category in the sample (N_2017_ = 605; N_2019_ = 634). In beverages where added sugar was combined with LNCS, the mean energy value almost halved (E_2017_: 92.8 kJ/100 mL; E_2019_: 96.2 kJ/100 mL) compared to only sugar-sweetened beverages. As expected, the lowest mean energy value was beverages that only contained LNCS (E_2017_: 22.3 kJ/100 mL; E_2019_: 14.3 kJ/100 mL).

Soft drinks had, surprisingly, lower energy values and sugar content in all four groups when compared to the whole sample, but we should note the high variability within this subsample (containing a great variety of different carbonated and non-carbonated drinks). The categories with the highest energy and sugar content were energy drinks with no LNCS, nectars with added sugar, and juices. We should note that some beverages were even over 200 kJ/100 mL. The lowest energy values and sugar content in all four groups were observed in sports drinks. The reduction in sugar content by replacing it with LNCS was most visible in sports and energy drinks, where all products with added LNCS (but no added sugar) had zero total sugar content. Similarly, the amount of sugar in soft drinks with LNCS was negligible (0.1 and 0.2 g per 100 mL in the years 2017 and 2019, respectively). Although the use of LNCS corresponds with lower mean energy content in most categories, this effect was less pronounced in nectars.

The difference in mean energy and sugar content in the selected categories of non-alcoholic beverages between the years 2017 and 2019 was in most cases not significant. The only significant difference was observed in energy drinks with added sugar and LNCS, where energy value and sugar content were significantly higher (ΔE: 81.2 kJ/100 mL; Δsugar: 4.6 g/100 mL; *p* < 0.01). Although the increase in the proportion of beverages with LNCS between 2017 and 2019 was not statistically significant (Table 1), we observed that the average sugar content of the whole sample decreased by 0.4 g per 100 mL (*p* = 0.03).

## 4. Discussion

To our knowledge, this was the first study on the use of LNCS in non-alcoholic beverages in the Slovenian food supply. The results showed that 13.2% of non-alcoholic beverages in 2017 and 15.5% in 2019 contained LNCS. This indicates a slight, but non-significant (*p* = 0.12) increase in the use of LNCS, over the course of two years. Comparison of our results with similar studies showed that the use of LNCS in non-alcoholic beverages in Slovenia was relatively low when compared to Latin America (>40%) [15], USA (23.9%) [26], or Spain (39.2%) [10]. A lower prevalence of LNCS use in non-alcoholic beverages has been reported only for New Zealand (8.7%) and Australia (2.3%) [26]. Even though these results show specific patterns in individual countries, we should note that the data collection took place in different ways, making comparison difficult.

Categories with the highest presence of LNCS were sports drinks and energy drinks, which is similar to findings in the study Dunford et al. (2018) [26]. This could indicate that consumers are seeking alternative products, particularly in these categories and/or that the technological application of LNCS is more manageable in these types of beverages [26]. Energy drinks have been associated with higher sugar content than other non-alcoholic beverages, and studies show that their excessive consumption presents a possible health risk. Using LNCS could be one of the possibilities to ensure the desired stimulant (from ingredients such as caffeine and taurine) effects of such beverages while reducing the negative effects of sugar by reducing the sugar content [31]. On the other hand, the reason for the high proportion of LNCS products in sports drinks could be the fact that the sample was mostly represented by isotonic drinks. Isotonic drinks typically have around 4–8 g of sugar per 100 mL [32]. Since average sugar content in other non-alcoholic beverages is higher, producers could add LNCS for additional sweet taste and better sensory acceptability by consumers. A lower proportion of LNCS use was observed in nectars and juices. We should note that according to local regulations [29], the use of any kind of LNCS is not allowed in juices, and it was, therefore, expected that we would not find such products.

Most beverages with added LNCS contained more than one LNCS, and only sucralose and steviol glycosides were used as sole sweetener. A similar finding was presented in the study from Chile [15]. One of the main reasons for this could be the taste of intensive sweeteners, which tend to have metallic or bitter after taste. With mixing LNCS the taste can improve, and the sweetness in some cases intensifies, which also helps lower the cost of LNCS. Steviol glycosides also have a bitter aftertaste, but mixing this sweetener with other LNCS is less common. This may be due to their natural origin (at least from a consumer’s perspective) and common marketing as a “natural sweetener”, even though this is not in line with the regulations. On the other hand, sucralose tends to have minimal after taste, and its taste among intensive sweeteners is the most similar to sucrose [15,33].

Our study showed that the most used LNCS in 2019 were acesulfame K and sucralose, similar to studies from Ireland [34] and Italy [35]. In Spain [10] and the USA [36], the major LNCS were acesulfame K and aspartame, similar to findings in Norway where they pointed out that in beverages, these LNCS are used mostly in combination [37]. A typical combination of acesulfame K and aspartame was also observed in our study. Chile reported on the high use of sucralose and steviol glycosides, both alone and in combination with other LNCS [15]. Assessments on dietary intake of the most common LNCS in Italy and Ireland showed that intakes in the general population were well below relevant ADI values for all age groups [34,35]. Chile has a greater concern with exceeding ADI values due to the increased consumption of LNCS and more frequent use of LNCS with lower ADI values, such as steviol glycosides [15].

The main difference between 2017 and 2019 was the increased use of sucralose and decreased use of aspartame. The use of sucralose was exceptionally high in energy drinks, where almost all new products that came to the market from 2017 to 2019 contained sucralose. Decreased use of aspartame could be due to its poorer temperature stability or because it is a source of phenylalanine and therefore not suitable for people with phenylketonuria. On the other hand, sucralose tends to have better temperature stability and, as mentioned before, has better sensory characteristics, which could be the reason for a significant increase in its use [33]. Intensive sweeteners were used in most cases, while polyols were found in just a few products. Polyols are not energy-free and provide 10 kJ/g compared to the 17 kJ/g provided by sugar. Polyols also have lower sweetness intensity, laxative effects, and some legal restrictions, so their use in beverages is less common, and in most cases, they are used as flavor enhancers [29,33]. In 2019 we also found two additional LNCS in non-alcoholic beverages, although they were only found in a few products (neohesperidin DC in five products and xylitol in one). This could indicate that manufacturers are looking for new ways to provide their products’ unique flavor [26]. These two LNCS are also known as good sweetness enhancers and can improve the overall taste of the beverage, which is a good option for reducing the cost of beverages.

Considering that manufacturers are using LNCS specifically to reduce the content of sugars in their products [38], lower energy and sugar content was expected for beverages sweetened with LNCS. The results showed an almost half reduction in the energy value of beverages that contained both added sugar and LNCS, compared to beverages with only added sugar. In beverages sweetened only with LNCS, the difference was even greater. These reformulation activities are particularly important if the purchase of LNCS containing products is also increasing, as was reported in the USA [39]. With an increased market share of the low-sugar product, the total sugar intake from beverages can decrease considerably. A recent study on prepacked products across four countries (Australia, Mexico, New Zealand, and the USA) revealed that products with LNCS had on average 8.7 g lower sugar content per 100 mL compared with products with no added LNCS [26]. Even though the sugar content of most non-alcoholic beverages in Slovenia is still high and sugar-sweetened beverages still represent more than half of the sample, our study showed that average sugar content decreased from 2017 to 2019 (0.4 g per 100 mL; *p* = 0.03), which could also be due to increased use of LNCS. This observation should be re-visited with a longer-term study, preferably with consideration to sales data. In our previous study, when the sale-weighing approach was also employed, we did not observe improvements in the sugar content in beverages between 2015 and 2017 [23].

An interesting observation in this study was that juices had an above-average energy value compared to other beverage categories. Consumers often see 100% juices as a healthier alternative to other sugary drinks. However, fruit juices commonly contain higher amounts of sugar than beverages that contain added sugars. Consequently, consuming large amounts of juices is also not recommended [40]. Fruit nectars were also a category with quite high energy and sugar content; even products with added LNCS were usually still high in sugar. This observation is related to the mandatory percentage of fruit content in nectars that naturally contain sugar [29].

Free sugars are problematic in the modern diet, and non-alcoholic beverages are one of the major sources. In Slovenia, soft drinks represent about 28% of all free sugars sold in grocery stores [23]. LNCS in beverages could help reduce free sugar intake while creating new potential risks to human health [17,18]. As the consumption of non-alcoholic beverages is high among children and teenagers [2], we can expect that increased use of LNCS, will lead to increased intake of LNCS in these groups. This opens a new issue since children can, in some cases (e.g., diabetics), exceed ADI values of some LNCS [15,17,41]. Some studies also suggest that LNCS consumption in children could reduce sensitivity to sweet taste and consequently to poorer eating habits later in life [42]. Even for adults, products with LNCS are not always the best choice. Non-alcoholic beverages often contain added acids, negatively affecting health by contributing to dental caries [43].

The sugar tax and related reformulation of products could be connected with the increased use of LNCS. Chile, Mexico, and partly the United States are countries with both high LNCS presence and established sugar tax [2,15,26]. These changes are clearly visible in Latin American countries, where excessive sugar intake and related diseases have been a major health problem [44]. After introducing a sugar tax, which has been shown as a successful measure for reducing sugar intake [45], the use of LNCS has risen sharply. The issue of LNCS overuse has already been highlighted in Chile, where LNCS can be found in all food categories, including baby food, which could lead to exceeding the ADI value, particularly in sweeteners with low ADI levels, such as steviol glycosides [15]. In Slovenia, there is no such legislation that would require sugar reduction. There are however, initiatives and (voluntary) responsibility pledges and commitments on sugar reduction, which show some results [23], and the risk of excessive sugar intake is also occasionally communicated in mass media, contributing to the education of consumers [46]. These activities could further influence the proportions of beverages with added LNCS. We need to keep this in mind when interpreting this study’s results and comparing them with other countries. It should be highlighted that only regular monitoring of the food supply will enable identification of changes in the market and any changes can be further correlated with health benefits and potential risks related to the use of LNCS. Reliable data on the composition and consumption of branded foods in the food supply is essential for the conduct of high-quality dietary surveys. A key output of the FNS-Cloud project is to enable easier access to datasets that can provide the data needed for such dietary monitoring studies.

A key strength of the present study was using a representative sample of non-alcoholic beverages, which included major retailers that represent the majority of the market share for branded foods. The sample consisted of beverages for two different time points, which enabled identifying current trends and changes in the food supply. Considering that Slovenia is part of the shared European Union marketplace and that many beverages in the food supply are brands of multinational companies, this study also offers insights into developments in the larger European food market. One possible limitation of our study is relying on the information on food labels and not chemical analysis when dealing with energy, sugar content and presence of LNCS. However, considering that food labelling is carefully regulated, we believe that in general the labelled composition of foods reflects the actual composition very well. A further limitation of this study is also not having data about the concentration of specific LNCS in products because food labelling only provides qualitative information on their usage. We therefore only investigated trends and changes in the use of LNCS but were unable to estimate exposures to LNCS through beverages, for which samples would need to be subject to laboratory analyses.

Further studies on LNCS should focus on this area, as together with current data they would provide a comprehensive insight into the use of LNCS. One of the limitations is also the two-year difference between the samples, as the market does not respond to changes quickly. Furthermore, the use of market-shares for specific foods would enable deeper analyses and identifications of trends for market-leading products.

## 5. Conclusions

We established that LNCS are present in 15.5% of non-alcoholic beverages and that their use slightly increased (*p* = 0.12) since 2017 (13.2%). The use of LNCS significantly increased only in energy drinks (*p* < 0.01), where most new products in 2019 contained LNCS. Comparison with other countries showed that the presence of LNCS in non-alcoholic beverages is relatively low and that there is still space for new products with added LNCS. The major LNCS in 2017 were acesulfame K and aspartame, while in 2019, the second most used LNCS was sucralose. As expected, the use of LNCS in beverages was associated with lower energy and sugar content. Since non-alcoholic beverages are one of the major sources of free sugar, LNCS in these products have the potential to reduce free sugar intake, so their further increase in the following years is expected. This food supply monitoring study will provide baseline data for tracking further changes in the food supply, which could also affect risk assessment scenarios used to determine safe levels of LNCS in these food categories. It should be emphasized that LNCS are just one option for sugar reduction and that producers should also be encouraged to reformulate products without using LNCS.

## Figures and Tables

**Table 1:** Sample description and the use of low and no-calorie sweeteners (LNCS) in non-alcoholic beverages in 2017 versus 2019 (N—number of all products; % diff.—% difference; ns—non-significant).

	2017		2019		*Z*–Test Statistics	
	Total	Added LNCS	Total	Added LNCS	Added LNCS	
	N (%)	N (%)	N (%)	N (%)	% Change	*p*-Value
Total	1043 (100)	138 (13.2)	1221 (100)	189 (15.5)	17.1	ns
Soft drinks	555 (53.2)	93 (16.8)	601 (49.2)	118 (19.6)	17	ns
Juices	267 (25.6)		330 (27.0)			
Nectars	135 (12.9)	16 (11.9)	158 (12.9)	11 (7.0)	−40.9	ns
Energy Drinks	65 (6.2)	11 (16.9)	110 (9.0)	46 (41.8)	147	<0.01
Sports Drinks	21 (2.0)	18 (85.7)	22 (1.8)	14 (63.6)	−25.8	ns

**Table 2:** Frequency of different types of low and no-calorie sweeteners (LNCS) in beverages with LNCS in 2017 and 2019 (N—number of all LNCS products; CI—confidence interval; ns—non-significant).

	2017 (N = 138)		2019 (N = 189)		*Z*–Test Statistics
	N (%)	95% CI	N (%)	95% CI	*p*-Value
Sole LNCS	40 (29)	22.1–37.0	73 (38.6)	32.0–45.7	ns
Mix of LNCS	98 (71)	63.0–77.9	116 (61.4)	54.3–68.0	ns
Acesulfame K	89 (64.5)	56.2–72.0	105 (55.6)	48.4–62.5	ns
Aspartame	75 (54.3)	46.0–62.4	64 (33.9)	27.5–40.9	<0.01
Cyclamates	48 (34.8)	27.3–43.0	50 (26.5)	20.7–33.2	ns
Saccharin	43 (31.2)	24.0–39.3	41 (21.7)	16.4–28.1	0.05
Sucralose	23 (16.7)	11.4–23.8	72 (38.1)	31.5–45.2	<0.01
Neohesperidin DC			5 (2.6)	1.1–6.0	
Steviol glycosides	35 (25.4)	18.8–33.2	43 (22.8)	17.4–29.2	ns
Xylitol			1 (0.5)	0.1–2.9	
Erythritol	1 (0.7)	0.1–4.0	3 (1.6)	0.5–4.6	ns

**Table 3:** Comparison of energy and sugar content in non-alcoholic beverages in 2017 and 2019, based on the presence of added sugar and low and no-calorie sweeteners (LNCS) (N—number of all products; SD—standard deviation; ns—non-significant).

	Added Sugar	Added LNCS	2017	2019	Energy Value (kJ/100 mL)			Sugar Content (g/100 mL)		
					2017	2019	*p*-Value	2017	2019	*p*-Value
			N (%)	N (%)	Mean (SD)	Mean (SD)		Mean (SD)	Mean (SD)	
**Total**			1043 (100)	1221 (100)	152 (66.5)	147.9 (70.9)	ns	8.0 (3.6)	7.6 (3.8)	0.03
All	✖	✖	300 (28.7%)	398 (32.6%)	174.8 (69.1)	174.9 (67.6)	ns	8.3 (3.8)	8.2 (3.8)	ns
	✔	✖	605 (58.0%)	634 (52.9%)	161.8 (50.8)	159.0 (52.7)	ns	8.9 (2.7)	8.7 (2.8)	ns
	✖	✔	59 (5.7%)	97 (7.9%)	22.3 (37.9)	14.3 (27.9)	ns	1.0 (2.1)	0.4 (1.4)	ns
	✔	✔	79 (7.6%)	92 (7.5%)	92.8 (28.1)	96.2 (40.0)	ns	4.8 (1.5)	5.1 (2.3)	ns
Soft drinks	✖	✖	26	43	60.2 (2.5)	70 (55.4)	ns	2.5 (3.5)	2.4 (2.5)	ns
	✔	✖	436	440	146.3 (8.2)	142.5 (50.9)	ns	8.2 (2.7)	7.9 (2.7)	ns
	✖	✔	39	50	4.4 (4.8)	5.8 (8.0)	ns	0.1 (0.2)	0.2 (0.8)	ns
	✔	✔	54	68	90.4 (29.7)	87.5 (35.1)	ns	4.9 (1.6)	4.7 (2.0)	ns
Juices	✖	✖	267	330	186.7 (57.0)	190.5 (54.8)	ns	8.8 (3.3)	9.1 (3.1)	ns
	✔	✖								
	✖	✔								
	✔	✔								
Nectars	✖	✖	5	23	135 (108)	141.8 (75.0)		7.8 (6.6)	6.8 (4.3)	
	✔	✖	114	124	203.3 (29.1)	198.8 (28.6)	ns	10.6 (1.6)	10.4 (1.8)	ns
	✖	✔	10	6	104.7 (5.4)	116.5 (23.1)		5.6 (0.4)	5.2 (0.7)	
	✔	✔	6	5	125.5 (29.1)	117.2 (16.0)		6.5 (1.6)	6.0 (0.6)	
Energy drinks	✖	✖	2	2	240.0	203 (52.3)		13.0	11.4 (2.3)	
	✔	✖	52	62	203.8 (28.7)	203.1 (31.4)	ns	11.4 (1.6)	11.3 (1.8)	ns
	✖	✔	8	36	11.5 (4.7)	10.6 (7.2)	ns			ns
	✔	✔	3	10	72.7 (7.2)	153.9 (45.6)		3.7 (0.4)	8.3 (2.7)	
Sports drinks	✖	✖								
	✔	✖	3	8	102.7 (26.5)	104.5 (16.3)		5.1 (1.2)	5.3 (0.7)	
	✖	✔	2	5	2.5 (3.5)	4.6 (2.6)				
	✔	✔	16	9	92.1 (12.3)	86 13.7)	ns	4.2 (0.4)	3.9 (0.2)	ns

## Data Availability

The data presented in this study are available on request from the corresponding author.
